# Epigenetic Effects of Human Breast Milk

**DOI:** 10.3390/nu6041711

**Published:** 2014-04-24

**Authors:** Elvira Verduci, Giuseppe Banderali, Salvatore Barberi, Giovanni Radaelli, Alessandra Lops, Federica Betti, Enrica Riva, Marcello Giovannini

**Affiliations:** Department of Pediatrics, San Paolo Hospital, University of Milan, Via A Di Rudinì 8, I-20142 Milan, Italy; E-Mails: giuseppe.banderali@unimi.it (G.B.); salvatorebarberi@hotmail.com (S.B.); giovanni.radaelli@unimi.it (G.R.); alessandra_lops@hotmail.it (A.L.); fedi.betti@hotmail.it (F.B.); enrica.riva@unimi.it (E.R.); marcello.giovannini@unimi.it (M.G.)

**Keywords:** epigenetics, human milk, breastfeeding, genetic polymorphism, nutritional programming

## Abstract

A current aim of nutrigenetics is to personalize nutritional practices according to genetic variations that influence the way of digestion and metabolism of nutrients introduced with the diet. Nutritional epigenetics concerns knowledge about the effects of nutrients on gene expression. Nutrition in early life or in critical periods of development, may have a role in modulating gene expression, and, therefore, have later effects on health. Human breast milk is well-known for its ability in preventing several acute and chronic diseases. Indeed, breastfed children may have lower risk of neonatal necrotizing enterocolitis, infectious diseases, and also of non-communicable diseases, such as obesity and related-disorders. Beneficial effects of human breast milk on health may be associated in part with its peculiar components, possible also via epigenetic processes. This paper discusses about presumed epigenetic effects of human breast milk and components. While evidence suggests that a direct relationship may exist of some components of human breast milk with epigenetic changes, the mechanisms involved are still unclear. Studies have to be conducted to clarify the actual role of human breast milk on genetic expression, in particular when linked to the risk of non-communicable diseases, to potentially benefit the infant’s health and his later life.

## 1. Introduction

### 1.1. Beneficial Effects of Human Breast Milk

Breastfeeding and human milk are the normative standards for infant feeding and nutrition. Short-and long-term benefits of breastfeeding on health are documented [1,2]. Breastfeeding has been associated with a reduction in the incidence of gastrointestinal tract infections, respiratory tract infections, and otitis media [1,2]. The relationship of human milk feeding with a significant reduction in the incidence of necrotizing enterocolitis (NEC) has been suggested in preterm infants [3]. Protective effects are shown also in autoimmune disorders (celiac disease, type-1 diabetes) and inflammatory bowel disease [1,2]. Additionally there is extensive evidence that individuals who had been breast-fed or received human milk show lower risk of some non-communicable diseases in later life [1,2]. Indeed breastfeeding has been associated with lower risk of obesity, lower levels of arterial blood pressure, lower total-and LDL-blood cholesterol levels in adulthood, and lower risk of developing type-2 diabetes [1,2]. Furthermore consistent differences in neurodevelopmental outcome between breastfed and formula fed infants have been reported [1,2]. Evidence about the association between neurodevelopment and exclusive breastfeeding was provided by the cluster-randomized Promotion of Breastfeeding Intervention Trial (PROBIT) study [4]. Adjusted outcomes of intelligence scores were significantly greater in exclusively breastfed for three months or longer.

Human milk consists not only of nutrients, but also of biologically active compounds, which may play an important role in the health benefits associated with breast-feeding [1]. For example nutrition is one of many factors that affect brain development not only morphologically, but also for neurochemistry and neurophysiology. The NUTRIMENTHE (The Effect of Diet on the Mental Performance of Children) is a large collaborative European Project assessing the short- and long-term effects of specific nutrients and food components in early-post-natal diet on neurodevelopment through well-designed large-scale epidemiological studies [5]. The fatty acids provided in breast milk are thought to play a crucial role in this respect. Indeed, a recent review indicated that neurodevelopment and cognitive abilities may be enhanced by early provision of *n*-3 long-chain polyunsaturated fatty acids (LCPUFAs) through breast milk or docosahexaenoic acid (DHA)-fortified foods may improve neurodevelopment and cognitive abilities [6].

However nutrients should not be considered only as an energy source or as factors involved in the development of the organism. More recently molecular biology studies have shown that nutrients, either directly or by hormonal activity, are able to significantly influence the expression of genes [7]. Through the nutrigenomics it may be possible to identify mechanisms that underline individual variations in dietary requirements, as well as in the capacity to respond to food-based interventions [7]. In this way nutrigenomics may be able to provide personalized nutrition recommendations in order to improve the prevention and therapy of pathologies in which each would be predisposed [8]. The research, aimed to analyzing the influence of nutrients on health through nutrigenomics, find their basis on two observations:

1. The diet changes the gene expression (nutritional epigenetics).
2. The metabolic processes of nutrients may vary and affect the state of health depending on the individual genotype (nutrigenetics).

Nutrigenetics, a fundamental branch of nutrigenomics, has the purpose to identify the genetic variations influencing the way of digestion and metabolism of molecules introduced in the diet [9]. The analysis of the Single Nucleotide Polymorphisms (SNPs) has identified genetic variations linked to the risk of each individual. SNPs, single base-pair differences in DNA sequence, represent a primary form of human genetic variation. The presence of differences in genetic material due to a single nucleotide may explain not only the onset of certain pathological conditions, but also the different responses to nutrients/foods in the diet [10]. An example of application of the concepts of nutrigenetics concerns the relationship between the apolipoprotein E gene polymorphism and the diet. The subjects with the apoE gene promoter (−219G/T) polymorphism show higher levels of LDL cholesterol and apoB plasma concentrations after consuming a saturated fatty acids rich diet [11]. Therefore, the 219G/T polymorphism may partly explain the individual differences in response to the diet introducing the possibility of prevention of hypercholesterolemia and its complications consuming a saturated fatty acids poor diet in individuals with this particular genotype [11].

Current nutrition recommendations are based on estimated average nutrient requirements for a target population and aim to meet the needs of most individuals within a population but also to prevent non-communicable diseases [12]. In the case of specific genetic polymorphisms, personalized nutrition recommendations may be needed [13]. Nutrigenetics is a promising tool that may be important to refine current nutrition recommendations and to provide personalized recommendations in population subgroups.

### 1.2. Nutritional Epigenetics

If evidence suggests that genome may be able to influence the nutrition [9], nutrients may be able to regulate gene expression [14]. Genes and nutrition seem, therefore, to be in mutual relationship. The term epigenetics literally means on top of genetics and refers to processes that induce heritable changes in gene expression without altering the gene sequence [10]. Epigenetic processes are integral in determining when and where specific genes are expressed. Alterations in the epigenetic regulation of genes may lead to profound changes in phenotype. The major epigenetic processes are DNA methylation, histone modification, chromatin remodeling and microRNAs, although it is still debated if miRNA may be considered as an epigenetic phenomenon. To date, most studies on the effect of early-life nutrition on the epigenetic regulation of genes have focused on DNA methylation [15,16,17,18]. Methylation of the 5′ position of a cytosine within the genome occurs by the enzymatic family of DNA methyltransferases forming 5-methylcytosine (5-mC), that is present in an estimated 4%–6% of the cytosine bases within a human genome. Most of DNA methylation occurs within CpG dinucleotides, although methylation outside of the CpG context has been reported in human DNA in recent years [18]. The human genome contains about 30 million CpG dinucleotides that exist in a methylated or unmethylated state. Dense repeats of CpG nucleotides are called CpG islands and occur throughout the genome. Methylation of CpG islands located in the promoter region of a gene is usually inversely associated with transcription of that gene due to binding of methyl-CpG binding proteins, which recruit proteins to the promoter of the gene, thereby blocking transcription. Therefore, epigenetics, that is the inter-individual variation in DNA methylation patterns and chromatin remodeling, provide a potential explanation for how environmental factors (e.g., bioactive food components, nutrients, specific diets) can modify the risk for development of many common diseases [16,17]. Age, genetics, and environment may together interact to affect epigenetic regulation. The epigenetics determinants may interfere at any time during the life of the individual [18]. Several studies have shown that the environment and nutrition, at an early stage or at critical periods of development, may influence the expression of genes with short- and long-term effects on the organism [15,16,17].

Data obtained from animal models suggest that maternal malnutrition during pregnancy results in a retardation of growth but also in a modification of the expression of biochemical mechanisms related to the endocrinological and metabolic control [15]. Indeed, it has been showed that offspring of mothers in a protein-restricted diet, from conception throughout pregnancy, present an altered metabolic phenotype showing a number of features of human cardio-metabolic disease, including hypertension, increased fat deposition, impaired glucose homeostasis, dyslipidaemia and vascular dysfunction [16]. In rats, maternal protein-restriction seems to epigenetically program metabolism in the offspring. In pups whose mothers were fed a diet low in protein was observed a reduced methylation and increased expression of peroxisome proliferator-activated receptor a (PPARα) in the liver [18]. Similar results were seen for the glucocorticoid receptor gene [18]. More recently, a low protein maternal diets in pigs was shown to effect global DNA methylation in the newborn offspring through changes in DNA methyltransferase (Dnmt1, Dnmt2 and Dnmt3) expression in both the liver and skeletal muscle [18]. These findings may demonstrate the influence of the maternal diet on the pup’s fat and carbohydrate metabolism.

Human studies found that adult disease risk may be associated with adverse environmental conditions early in development. In particular, the risk of obesity and its associated conditions may be related to the timing of nutrient constraint during pregnancy [19]. Several studies, focused on individuals exposed to famine *in utero* which occurred in the Netherlands during the winter of 1944, presented evidences that individuals whose mothers were exposed to famine periconceptually and in the first trimester of pregnancy showed low birth weight compared with unexposed individuals and, as adults, exhibited increased risk of obesity and cardiovascular disease [20]. In addition, nutrition in early postnatal life may affect susceptibility to future obesity [21]. Early catch up growth in infants born preterm, who also have a reduced fat mass at birth, and who were formula fed show increased risk of cardio-metabolic disease in later life, including obesity [21]. The exact mechanisms underlying how early nutrition may cause programming of risk of non-communicable diseases are unknown, but are thought to be associated with altered development of organ structure or persistent alteration at cellular level [22]. Among proposed mechanisms, acute or persistently altered gene expression through a variety of epigenetic pathways may be included [22]. During *in utero* or early postnatal development, short-term changes through environmental influences could permanently change organ development at a time of extreme vulnerability or “plasticity” [22]. A recent study is the first example of an association between periconceptional exposure to environmental factors and DNA methylation in humans [20]. Individuals who were prenatally exposed to famine during the Dutch Hunger Winter had, six decades later, showed less DNA methylation of the imprinted Insulin Growth Factor (IGF) 2 gene compared with unexposed, same-sex siblings. The association was specific for periconceptional exposure, reinforcing that very early mammalian development is a crucial period for establishing and maintaining epigenetic marks [20]. Changes in epigenetic marks as differences in methylation of the IGF2 DMR (differentially methylated region) could affect the phenotypic expression and be associated with an increased risk of adult disease considering that IGF2 is a key factor in human growth and development [20].

These findings demonstrate that the prenatal and early postnatal periods have a critical role in the individual outcome, as Barker affirms: “Much of human development is completed during the first 1000 days after conception” [21]. At least epigenetics might partially explain the mechanism that delucidates the fetal “programming” [16,17].

### 1.3. Topic of Review

While beneficial effects of human breast milk on health have been recognized, it is, nowadays, debatable whether these effects may be mediated and/or linked with epigenetic processes. The aim of this paper is to discuss on this topic with respect to current evidence investigating the role of human breast milk and components in controlling epigenetic changes.

## 2. Epigenetic Effects of Human Breast Milk

Breast milk, with nutritional but also functional components, is a real biological system. According to present knowledge, it is associated not only with improved parameters of growth, but also with a better neuronal-behavioral development [1,23]. It is also associated with the prevention of some communicable and non-communicable diseases [1,2]. The source of these health beneficial effects may be the peculiar composition of breast milk, partly explained by epigenetic processes. Indeed, the relationship between nutrition in early life and genome may allow to understand the underlying mechanisms of disease that have high impact on individual health.

### 2.1. Neonatal Necrotizing Enterocolitis

Neonatal Necrotizing Enterocolitis (NEC) is a severe intestinal inflammatory disorder in newborns. Although the pathogenesis is not completely understood, NEC may be associated with an inappropriate innate immune and excessive inflammatory response of the immature intestine. It has been reported in several studies that the incidence of NEC was higher in formula-fed than in breastfed babies [24]. Recently, breastfeeding has been associated to 77% reduction risk in preterm infants compared to formula [1]. One explanation for the reduced incidence of NEC in infants fed with human milk is the enhanced production of secretory IgA (sIgA) in human milk feeding [25] that provides protection against pathogenic organisms. Indeed deficient IgA production in preterm neonates may facilitate bacterial translocation across the intestinal mucosa. Moreover, breast milk provides a multitude of proteins with anti-inflammatory properties, according to both *in vitro* and *in vivo* studies, such as anti-inflammatory cytokines (e.g., TGF β, transforming growth factor beta) [26]. However, an inappropriate and early alteration in composition of gut microbiota or an unfavorable balance between commensal and pathogenic bacteria (dysbiosis) has been strongly involved in the pathogenesis of NEC [25,27]. Full-term, vaginally born infants are completely colonized with a diverse array of bacterial families by the first year of life. The type of oral feeding (e.g., breast *vs*. formula feeding) may strongly influence short-term composition of infant’s gut microbiota [28]. At six months, weaning to a solid diet leads to complete colonization and the infants have a unique signature of microbiota with them throughout their lifetime. Appropriate colonization is influenced by the prebiotic effect of breast milk oligosaccharides. Breast milk has a large percentage of undigestible oligosaccharides (e.g., 8% of total calories), which function as prebiotics, providing substrate for the production of short-chain fatty acids, leading to the proliferation of health-promoting bacteria, such as *Bifidobacteria* and *Lactobacillus*. An association between the levels of secretory IgA in intestinal secretions and the number of *Bifidobacteria* in the gut at one month of age has been showed [29]. Therefore, human breast milk may have a role in preventing NEC with programming sIgA excretion through the influence on gut microbiota composition. Additionally, commensal bacteria may regulate the expression of genes important for barrier function, digestion, and angiogenesis. *In vitro* studies it has been demonstrated that many species of commensal bacteria may reduce the inflammatory response by inhibiting the nuclear factor kappa-light-chain enhancer of activated B cells (NF-κB) [25]. This is very important considering that the balance of pro- and anti-inflammatory signaling is critical in maintaining normal intestinal functions. Moreover, it has been found *in vitro* that human breast milk suppresses the interleukine (IL) 1-β-induced activation of the IL-8 gene promoter in human intestinal cells by inhibiting the activation of NF-κB [30]. Some investigations on the immunomodulatory role of human breast milk through regulation of gene expression lead to lactoferrin properties, an abundant breast milk protein [31]. Lactoferrin is able to bind proinflammatory bacterial DNA sequence (CpG motifs) in extracellular compartments and this binding seems to inhibit the CpG- motif DNA-induced activation of NF-κB-regulated genes, such as IL-8 and IL-12 in B cells [31]. Bacterial DNA CpG motifs may be present in the lamina propria and Peyer’s patches due to lysis of entheropathogens, and human milk lactoferrin may modulate immune responses concerning to lymphoid follicles of the infant intestine [31].

In conclusion results from literature suggest a possible direct and/or gut microbiota programming mediated epigenetics role of human breast milk in preventing preterm infants’ NEC by suppressing the NF-κB signaling pathway involved in the regulation of proinflammatory cytokines genes, as Interleukin-8.

### 2.2. Infectious Diseases and Disorders of the Immune System

The preventive effect on infections is one of the most important health benefits in relation to breastfeeding [1,2]. Breastfeeding is strictly associated with a lower risk of gastrointestinal infections and of acute otitis media [1,2]. Important breast milk components may influence infection susceptibility, such as anti-inflammatory cytokines and pathogen neutralizing secrectory IgA antibodies [26]. Recently, proinflammatory cytokine gene polymorphisms, such as TNF-α and Interleukin-6 gene polymorphisms (TNF-α^−308^ and IL-6^−174^), have been associated with increased risk for otitis media (OM) susceptibility [32]. Breastfeeding may protect against OM even when children are carriers of TNF-α^−308^ and IL-6^−174^ polymorphisms stressing the evidence that environmental factors, such as breastfeeding, may help to reduce occurrence of diseases also in genetically susceptible subjects [32].

Breastfeeding is associated with a reduction in the risk of childhood inflammatory bowel disease and of celiac disease of 31% and 52%, respectively, in infants who were exclusively breastfed at the time of gluten exposure [1]. This protective role may result from the interaction of immunomodulating effect of human milk and the underlying genetic susceptibility of the infant. In addition, the different patterns of gut microbiota composition in breastfed *versus* formula fed infants may be linked to the preventive effect of human milk, taking into account that gut microbiota may have a role in programming the immune phenotype [33,34]. As for NEC, the epigenetic role of human breast milk in preventing infectious diseases and disorders of immune phenotype, direct and/or gut microbiota mediated, may be linked to the expression regulation of proinflammatory cytokines genes.

### 2.3. Obesity and Related-Disorders

Breastfeeding, compared with formula feeding, has been associated with lower risk of being obese, lower risk of developing type-2 diabetes, lower total cholesterol and lower levels of arterial blood pressure [1,2]. The plausible mechanisms by which breast-feeding may show a protective role could be the nutrients composition of breast milk and a peculiar feeding behavior associated to breastfeeding. Indeed, lower protein and energy content in breast milk than formula, jointly with higher content of LCPUFA, cholesterol and non-digestible carbohydrate (substrate for beneficial strains, *Bifidobacterium* and *Lactobacillus* spp., growing in gut microbiota) may act synergistically, additionally with better hunger and satiety self-regulate of breast-fed infants (possibly modulated by some hormones and hormone-like compound, e.g., ghrelin and leptin), to determine healthier outcomes [35].

The risk of developing obesity depends on the interaction between genotype and individual lifestyles, and also environment and nutrition during fetal life and in the early ages of life are very important [19,20,21]. The epigenetic regulation of specific genes may also become crucial in determining the individual risk for obesity. The peroxisome proliferator-activated receptor-γ (PPARγ2) transcription factor is primarily expressed in adipocytes [36]. It is a member of the nuclear hormone receptor family, influencing whole body energy homeostasis via three main metabolic pathways: adipocyte differentiation, insulin sensitivity, and lipoprotein metabolism [36]. Out of several variants identified in the PPARγ2 gene, the most common is the Pro12Ala substitution at codon 12 [37,38,39]. This polymorphism has been shown to be associated with reduced ability to transactivate responsive promoters and, in adults, with higher BMI, waist circumference, and obesity risk [37,38,39]. In a recent study the PPARγ Ala12 allele was associated with higher adiposity indexes (BMI, waist circumference, and the sum of skinfolds) in adolescent who had not been breast-fed [40]. However, this association was not seen in Ala 12 carriers who had been breast-fed (even for a short period). This result may suggest both that breastfeeding may have beneficial effect on the obesity risk later in life in genetically predisposed groups [40] both that breast milk may have an epigenetic effect associated to the adiposity and related-disorders development. Indeed, it has been described that breast milk supplies factors, such as prostaglandin J2, arachidonic acid derivative, natural PPARγ ligand [40]. The decrease in PPARγ2 transcriptional activity observed in Ala12 allele carries could be, therefore, compensated for by breast milk. In experimental studies PPAR-γ expression has been considered important also in reducing liver fibrogenesis [41]. Indeed, in nonalcoholic fatty liver disease (NAFLD), an obesity-related disorders, PPAR-γ expression has been suggested as possible target of therapeutic approachs [42]. Recently, some authors have hypothesized a beneficial effect of breastfeeding duration on progression of NAFLD, particularly on non-alcoholic steatohepatitis (NASH) and liver fibrosis [43]. In addition, for this protective role of breastfeeding may be considered an epigenetic mechanism. Long-chain polyunsaturated fatty acids of the *n*-3 series, especially DHA could act as activators of PPAR (α and γ) implicated in protection against fibrosis [42]. DHA enriched diet has been found to reduce the risk of liver steatosis in animals by down-regulating the liver lipogenic and cholesterol biosynthesis [44].

The possibility that early infant feeding has long-term effects on blood cholesterol levels is supported by many studies [1,2]. Breastfeeding seems to be associated with increased mean total cholesterol and LDL cholesterol levels in infancy but lower levels in adulthood [45,46]. Dietary cholesterol intake seems to be the main determinant of total cholesterol level in infancy [46]. The high cholesterol content of breast milk may well be responsible. High cholesterol intake in infancy may reduces endogenous cholesterol synthesis, probably by down-regulation of hepatic hydroxymethyl glutaryl coenzyme A (HMGCoA) reductase [45,46]. Additionally it has been showed that *n*-3 LCPUFA may modulate the HMGCoA reductase expression in rats [47]. This epigenetic mechanism, high cholesterol content in breast milk and down-regulation of HMGCoA reductase, might be further studied.

Moreover the contribution of the gut microbiota, such as dysbiosis, to the development of obesity and obesity-related disorders, including diabetes, atherosclerosis, and NAFLD, is becoming clear [48,49], even if more studies are needed. From this view point the beneficial effects of breastfeeding on obesity and related alterations could be mediated partly by programming a healthier composition of gut microbioma, inducted by some breast milk components (nondigestible oligosaccharides).

### 2.4. Cancer

Breast milk is functionally positive not only for the child but also for the mother.

Considering the proven existence of an inversely correlation between breastfeeding duration and breast cancer risk [50], an important case-control study, examined the relationship between breast-feeding and breast cancer risk among women who carried deleterious mutations in the BRCA1 or BRCA2 gene [51]. The results showed that women with deleterious BRCA1 mutations who breast-fed for a cumulative total of more than one year, presented a statistically significantly reduced risk of breast cancer than those who did not breast-feed their children [51]. No association were found between breast cancer risk and breast-feeding for women with BRCA2 mutations, probably because of the small size of sample. Breastfeeding may reduce the risk of breast cancer both directly by altering the hormonal milieu that indirectly by delaying the re-establishment of ovulation in the way to induce changes in mammary gland differentiation [51]. A recent study showed that *in vitro* DHA, a natural ligand of peroxisome proliferator-activated receptors, is able to modulate PPARβ mRNA expression inhibiting breast cancer cell growth and mammary tumor growth [52].

Further studies are needed to find a possible epigenetic link between human milk component and prevention of breast cancer.

## 3. Breast Milk and Environmental Factors: Smoking as Epigenetic Factor

Maternal smoking during pregnancy has been associated with a reduced content of *n*-3 LCPUFA in breast milk, particularly concerning DHA, reducing the intake of these key nutrients to the infants [53]. The exposure to cigarette smoke may negatively affect the synthesis of *n*-3 LC-PUFA from the precursor in mammary gland cells. Indeed, *in vitro* studies, showed that there is a dose-dependent relationship between smoking and the inhibition of both the conversion of the precursor alpha-linolenic acid to *n*-3 LC-PUFA and of the D5 desaturation step [54].

## 4. Discussion

Although the different epigenetic mechanisms involved remain unclear, the benefits of breastfeeding against NEC, infectious diseases, obesity and related-disorders, and cancer might be partly explained by the epigenetic model. Breast milk, modulating gene expression without changing the nucleotide sequence of DNA, might positively modify the phenotype and the outcome even if there is a genetic predisposition for the development of diseases. Possible epigenetic effect of human breast milk components on a child’s health outcomes are summarized in Table 1. However, further studies are warranted to provide more explanations about the direct relationship between human breast milk and components and gene expression, particularly regarding prevention of non-communicable disease in infants carrying genetic polymorphisms associated to risk of these diseases. Considering the important role of human milk in the development of later diseases, breastfeeding support and promotion should be a priority for each community. Indeed the individualized strategy according to nutrigenomics perspective should not distract from a global strategy.

**Table 1:** Epigenetic effect of human breast milk components on the child’s health outcomes.

Human Milk Component	Prevention of	Gene (Expression)
Lactoferrin	NECDisorders of immune system	NF-kB (reduced) ^(a)^
Prostaglandin J	Obesity and related-disorders	PPARγ (increased) ^(b)^
LCPUFA *n*-3	NAFLD	liver lipogenic and cholesterol byosynthesis enzymes (reduced) ^(a)^
	Progression of NAFLD	PPAR α and γ (increased) ^(b)^
	High blood total cholesterol in adulthood	HMGCoA reductase (reduced) ^(a)^
Cholesterol content	High blood total cholesterol in adulthood	HMGCoA reductase (reduced) ^(b)^
Undigestible oligosaccharides	Gut dysbiosis and related alterations (NEC, infectious diseases, disorders of immune system, obesity and linked disorders)	action on expression of different genes (e.g., NF-κB) ^(b)^

^(a)^ proved *in vitro* and/or animals ^(b)^ hypothesized in humans.

## 5. Conclusions

The current aim of nutrigenetics is to personalize nutritional practice according to genetic variations that influence the way of digestion and metabolism of nutrients introduced with the diet. Nutritional epigenetics concerns knowledge about the effects of nutrients on gene expression. Human breast milk is well-known for his properties to prevent several communicable and non-communicable diseases in infancy and in adult life. Breastfed children may have lower risk of NEC, infectious diseases, later obesity, and related disorders, and breastfeeding mothers may have lower risk of breast cancer, even if a genetic predisposition for the development of these diseases is present. The source of these health beneficial effects may be the peculiar composition of breast milk. The benefits associated to breastfeeding might be partly explained by epigenetic processes. However, the different epigenetic mechanisms involved are still unclear. Further studies are warranted to provide more explanations about the relationship between human breast milk and gene expression, particularly regarding prevention of non-communicable diseases to potentially benefit the infant’s health and his later life.
